# Trans* People Experiencing Domestic and Intimate Partner Violence: Insights from Professionals Within Portugal’s National Support Network

**DOI:** 10.3390/healthcare14101390

**Published:** 2026-05-19

**Authors:** Luiza Andrade, Pedro Alexandre Costa

**Affiliations:** 1 Faculty of Psychology and Education Sciences, University of Porto, 4200-135 Porto, Portugal; luu29andrade@gmail.com; 2 Center for Psychology at University of Porto (CPUP), Faculty of Psychology and Education Sciences, University of Porto, 4200-135 Porto, Portugal

**Keywords:** gender violence, transgender, domestic violence, RNAVVD

## Abstract

**Highlights:**

**What are the main findings?**

- Family violence affects the autonomy of trans* youth, resulting in unemployment and lack of access to housing.
- Interventions with trans* individuals are insufficiently covered in mandatory training for professionals assisting victims of domestic violence.

**What are the implications of the main findings?**

- Public policies should promote adequate social responses and strengthen trans* people’s rights by ensuring that services address their specific psychosocial needs.
- Professionals highlight the need to reassess mandatory domestic violence training and promote ongoing professional development so generalist services can better support the trans* population.

**Abstract:**

**Background/Objectives**: Despite legal progress and achievements regarding trans* rights in Portugal over recent decades, trans* individuals still face high levels of violence and discrimination, especially within family and intimate relationships. This qualitative study aimed to explore the experiences and perspectives of professionals working in the domestic violence field with the trans* population and service provision within the National Support Network for Victims of Domestic Violence (RNAVVD). **Methods**: Semi-structured interviews were conducted with eight participants, including psychologists, social workers, and program directors from organizations supporting victims of domestic violence in Portugal. Data were analyzed using Codebook Thematic Analysis to identify themes, resulting in two main themes: (1) Experiences in Working with Victimized Trans* Individuals; and (2) Framework of Portugal’s National Support Network. **Results**: The results showed that trans* individuals face significant vulnerabilities due to family and intimate partner violence, systemic discrimination, and inequalities in essential services. Young trans* individuals are seen as being particularly at risk due to the impacts of violence and lack of family support on their autonomy, and additional barriers to entering the labor market. Participants also identified barriers faced by this population when trying to access victim support services (e.g., lack of specialized training and low availability of specialized and culturally competent services), while highlighting efforts by LGBTQIA+ services to meet their psychosocial needs. **Conclusions**: In conclusion, public institutions must address the specific needs of trans* individuals by developing policies and services that adopt a cross-sectoral, intersectional approach across society.

## 1. Introduction

While the Portuguese Parliament currently recognizes trans* people’s right to self-determination, gender identity was not recognized in the country’s legislation until 2011. The term trans*, with an asterisk, has been used to express a critical perspective on the sometimes-shared needs of trans* individuals, while also acknowledging the heterogeneity of their lived experiences and identities. Throughout this study, the term is adopted to refer to individuals who identify with a gender different from the one assigned at birth, including those who identify outside the gender binary. This meant that trans* people seeking to change their official documents had to sue the State, facing proceedings that could last several years and often included court-mandated requirements that infringed upon their human rights, such as proof of sterilization, divorce, and medical interventions [1,2]. In 2011, the Law No. 7/2011 established an official procedure for adults, but it required a clinical diagnosis of Gender Identity Disorder, and excluded minors and non-nationals [1]. In 2018, the Law No. 38/2018 further advanced trans* people’s rights by establishing legal gender recognition based on self-determination and extending eligibility of social and legal transition to 16- and 17-year-olds with parental consent. Despite these advances, non-binary identities remain unrecognized, and non-nationals are still not fully included [1].

Notwithstanding these legal advances, evidence suggests a persistent gap between formal rights and individual everyday experiences, with LGBTQIA+ individuals in Portugal continuing to report discrimination across multiple areas of their daily life [3]. Data from the EU LGBTQI Survey II indicate that trust in the Portuguese government’s efforts to combat prejudice declined from 56% in 2019 to 38% in 2023 [4]. Portugal also fell from 10th to 11th place in the Rainbow Map, which ranks 49 European countries on LGBTQIA+ rights [5].

Furthermore, research on the lived experiences of trans* individuals in Portugal remains limited, especially regarding discrimination and violence, due to a lack of comprehensive national data on LGBTQIA+ populations [1,2]. Current knowledge relies heavily on reports from LGBTQIA+ organizations and international agencies, which indicate that Portugal reports some of the highest levels of discrimination against trans women in Europe [4]. National studies further show that trans* people face heightened vulnerability due to intersecting forms of violence, discrimination, and structural inequality, which restrict access to basic services and support networks [6,7]. Within this context, professionals working in victim support services play a crucial role in the recovery process of victimized individuals, and their training and the development of cultural competencies are key elements in breaking this cycle of violence.

### 1.1. Domestic and Intimate Partner Violence

To facilitate the understanding of the present study, some key concepts require clarification. Domestic violence is defined in the Portuguese Penal Code as a public crime and refers to a pattern of ongoing violent behavior or coercive control exercised, directly or indirectly, against individuals who share the same household or who, even if not cohabiting, are current or former partners or family members [8]. Intimate Partner Violence (IPV) emerged as a concept that expands the understanding of domestic violence beyond marital contexts, acknowledging that violence may occur across diverse forms of intimate relationships [8]. When perpetrated against LGBTQIA+ individuals, it refers to violent behaviors directed toward a spouse, partner, child, parent, or grandparent who identifies as LGBTQIA+ or is perceived by others as being LGBTQIA+ [9]. The conceptual definitions presented above are adopted in this article and underpin its analytical framework.

Data on domestic violence and IPV show that these are serious public health issues that disproportionately affect women. The World Health Organization estimates that between 2000 and 2018, around 27% of women aged 15–49 experienced some form of IPV [10]. In Europe, EIGE [11] reports that among women respondents (*n* = 114,023), almost 31% experienced physical or sexual violence, with a prevalence of 25% in Portugal. Additionally, the latest Annual Internal Security Report in Portugal [12] indicates that 30,221 domestic violence reports were made in the last year, with 85.5% involving IPV. Victims were primarily women (67.9%), and perpetrators were mostly men (78.2%).

While these general statistics highlight the gendered nature of IPV, data specific to LGBTQIA+, and particularly trans* individuals, in Portugal remains scarce, making it difficult to estimate its prevalence among LGBTQIA+ individuals [2]. Although domestic violence and IPV are gendered phenomena, with girls and women being disproportionally victimized, several authors have also highlighted a cisheteronormative bias in dominant narratives surrounding these forms of violence, which contributes to the invisibilization of the experiences of individuals with diverse sexual orientations and gender identities [13,14]. Nevertheless, existing research shows that LGBTQIA+ people, especially trans* individuals, face comparable or higher victimization risks within intimate relationships [15,16,17].

For trans* people, family and intimate relationships are the contexts in which they face a greater risk of victimization [2]. LGBTQIA+ youth are particularly vulnerable to family abuse, including being forced out of their homes and having family ties severed. Additionally, trans* people face increased risks of suffering from psychological and physical abuse, including microaggressions [18]. Within intimate relationships, a U.S. study [15] with adult LGBTQIA+ individuals (*n* = 1139) found that about 20% had experienced IPV, with trans* participants (*n* = 112) reporting higher rates. Another study [17] with cisgender and trans* individuals (*n* = 3560) showed that trans* people faced greater risks of physical violence and IPV in the previous year, and that trans men and non-binary people were more likely to experience sexual violence than were cisgender women.

Research consistently shows that trans* populations face heightened risks of adverse physical and mental health outcomes, including social isolation, depression, anxiety, post-traumatic stress disorder, and suicide, as well as increased psychosocial vulnerability, such as unemployment, exposure to violence, and homelessness [19]. These outcomes are closely linked to experiences of discrimination across key social contexts, including education, employment, and healthcare, which have been associated with elevated depressive symptoms and suicidal ideation [18].

In the context of domestic violence and IPV, trans* individuals are particularly affected by intersecting forms of violence, discrimination, invisibility, and isolation, resulting in psychological distress, gender dysphoria, physical assaults, self-harm, and suicide attempts [7]. A systematic review and meta-analysis [20] further elucidate the consequences of intimate partner violence among trans* individuals, identifying long-term psychological impacts such as cumulative trauma, hypervigilance, fear of future relationships, and a wide range of mental health difficulties. This systematic review also highlights a recurrent tension between the desire for intimacy and the need for safety, whereby avoidance of intimate relationships emerges as a strategy to prevent revictimization but often leads to increased social isolation. Significantly, the internalization of structural transphobia and transphobic manipulation by abusive partners may reinforce barriers to leaving violent relationships and contribute to long-term relational avoidance and social withdrawal.

### 1.2. Help-Seeking, Barriers, and the National Support Network

An essential part of recovery for people who have been victimized by domestic violence and IPV is seeking help from support networks, which can be formal (e.g., institutions, victim support services, healthcare providers, police, etc.) or informal (e.g., family, neighbors, coworkers, etc.). Asking for help usually improves victims’ safety and health outcomes; however, they often face barriers that prevent them from accessing support services, such as fear of the abuser, fear of not being believed by others, lack of awareness of community resources, and stigma surrounding domestic violence [21].

For LGBTQIA+ people, additional barriers can further discourage reporting and help-seeking through formal support networks, including: limited availability of specialized and culturally competent services, economic dependence on aggressors, fear of being thrown out of home, challenges in achieving autonomy, concerns about having to reveal their LGBTQIA+ identity, and concerns about experiencing discrimination because of it [13,20,22]. Furman et al. [13] further emphasize that LGBTQIA+ people often experience social invisibility and that domestic violence is commonly framed through a heteronormative lens, which can make recognition of domestic violence within this population difficult. Specifically for trans* people, research indicates a lower likelihood of seeking formal support, primarily due to fear of gender identity-based discrimination [20,22]. The inability to seek support from families, which often reject them, and the fear of reporting cases to the police or healthcare professionals significantly increase barriers for this population [20].

Acknowledging these barriers and the need for targeted interventions, the Portuguese government established specialized services for the LGBTQIA+ community in 2016, provided by three institutions: ILGA-Portugal and Casa-Qui in Lisbon, and Plano i in Porto [23]. In 2018, the first emergency shelter for this population, Casa Arco-Íris, was inaugurated and is managed by Plano i. These three services are part of Portugal’s National Support Network for Victims of Domestic Violence (RNAVVD), which encompasses the public administration responsible for citizenship and gender equality (CIG), Social Security, shelter services (e.g., shelters and emergency accommodation), and victim support services. The RNAVVD covers the entire territory and comprises approximately 200 structures.

Despite the importance of establishing specialized services, several challenges have been reported regarding their response capacity. In addition to being limited in number, these services lack qualified personnel and specific technical resources necessary to provide effective care and intervention for all victimized individuals [7]. Furthermore, the existence of only one emergency shelter response for LGBTQIA+ people, combined with the absence of a shelter specifically for this group, creates significant barriers to access. Because victimized individuals may remain in the emergency facility for up to nine months, the facility is frequently at full capacity, thereby limiting its ability to admit new cases [24].

### 1.3. Studies with Professionals Working in the Field of Domestic Violence

Recent studies have examined professionals’ experiences working with LGBTQIA+ individuals who have experienced domestic violence and/or intimate partner violence. Two qualitative studies from the United States [13,25] with professionals (*n* = 10) reveal a lack of specialized LGBTQIA+ training and feelings of unpreparedness, especially when working with trans* individuals, who face high levels of discrimination and exclusion in services. Barriers include fear of discrimination, the absence of inclusive protocols, limited programs, and insecure funding for LGBTQIA+ organizations. Both studies emphasize the need for staff training and the development of inclusive, culturally competent protocols.

In Portugal, Neves et al. [7] conducted a mixed-methods study with four focus groups (*n* = 24), 11 interviews (*n* = 121), and online surveys (*n* = 205) involving professionals from the public administration, NGOs, and LGBTI+ groups. Results showed that the Central Public Administration is perceived as hostile and discriminatory toward LGBTQIA+ people, highlighting the need to adapt training curricula. Trans* and intersex individuals face high levels of discrimination, violence, and invisibility. Although 75% of participants received domestic violence training, only 36% received LGBTQIA+-specific training. The study concluded that violence and discrimination, compounded by intersectional factors such as ethnicity, nationality, and migratory status, increase vulnerabilities among victimized LGBTQIA+ individuals.

Despite differences in study methods and contexts, the reviewed research identifies shared concerns among professionals working with domestic violence and intimate relationships, mainly regarding services for LGBTQIA+ and victimized trans* individuals. The lack of knowledge and training on LGBTQIA+ issues underscores the need for better capacity building and culturally competent protocols. These studies also highlight the increased vulnerability of trans* individuals and barriers to support.

Although research on domestic violence and IPV affecting trans* individuals has increased in recent years, a scarcity of studies examining professional practices and institutional responses within national support systems remains. In the Portuguese context, little empirical work has focused on how services address the specific needs of trans* people, despite growing recognition of the importance of inclusive, coordinated, and specialized responses in the domestic violence field. Therefore, the present study aimed to examine the experiences of professionals working in the domestic violence sector who provide support to trans* individuals, as well as their perceptions of initiatives directed at this population within Portugal’s National Support Network for Victims of Domestic Violence (RNAVVD).

## 2. Materials and Methods

### 2.1. Participants

Eight professionals working in the field of domestic violence participated in this study. The majority (*n* = 5) were employed in specialized LGBTQIA+ services, whereas the remaining participants (*n* = 3) worked in ‘generalist’ services, i.e., in services that cater to the general population. To align with the study’s objectives, the following inclusion criteria were established: (i) professional experience providing services to individuals victimized by domestic violence and IPV; (ii) experience working with LGBTQIA+ individuals; and (iii) employment in a service that is part of the National Support Network (RNAVVD). All participants met these criteria and were included in the study.

Most participants used feminine pronouns (*n* = 7), whereas one participant used masculine pronouns. All participants worked in services located in the two major cities (Lisbon and Oporto). Participants’ ages ranged from late twenties to pre-retirement age. Regarding professional qualifications, most participants (*n* = 5) held a psychology degree, one held a degree in social work, and two held degrees in other fields (criminology and cultural studies) with specialized training in gender studies. Most participants completed the mandatory Victim Support Technician (TAV) training course at the time of data collection, with only one indicating ongoing completion of the requisite training. Professional experience ranged from approximately 1 to 27 years.

### 2.2. Materials

Materials for this study included an interview script, a consent form, and a debrief form. The script was pilot tested with one professional experienced in research and in supporting LGBTQIA+ individuals who had experienced domestic violence and IPV. Questions were organized into three sections: (a) professional background and services (e.g., education, types of services provided, targeted population); (b) service provision to trans* individuals (e.g., case experiences, specific needs, collaboration with other services, institutional practices); and (c) reflection on the National Support Network (e.g., service adequacy, barriers, and broader systemic needs). To ensure alignment with the study’s objectives, the script was reviewed and refined through meetings with the second author and in consultation with the contributing professional to incorporate practice-based insights. Prior to recruitment, a pilot interview was conducted to evaluate the script’s effectiveness and to familiarize the first author with the interview procedure.

### 2.3. Procedures and Ethical Considerations

Ethical approval was obtained from the FPCEUP’s Ethics Committee (reference no. 2024-10-15b). Participants were recruited using two nonprobability sampling methods: purposive sampling, in which 19 key institutions were selected (identified through a search in the Domestic Violence Resource Guide provided by CIG), resulting in a total of five participants; and convenience sampling, in which individuals with whom the researchers had direct contact were identified, resulting in the remaining three participants.

Semi-structured interviews were conducted by the first author between March and June 2025 via an online conferencing platform. With participants’ consent, interviews were audio-recorded and supplemented with field notes, with an average duration of approximately 60 min. In one case, two interviews were conducted with the same participant to allow for more in-depth exploration of topics that could not be addressed comprehensively in the initial interview.

Informed consent was reviewed and obtained by having participants sign a consent form. Participants were also invited to share their preferred pronouns for use during the interview. Each participant also received a debrief form after the interview, which included supplementary information on the study’s objectives, contact information of the research team, and relevant community resources. Participation was voluntary, and the right to withdraw at any time was guaranteed without justification. To ensure confidentiality and anonymity, interview audio files were securely stored, with access limited to the research team, and deleted after transcription. Identifiable information, such as names and speech characteristics, was removed from transcripts. Finally, participants’ names were replaced with pseudonyms and initials, which were used throughout this study.

### 2.4. Data Analysis and Reflexivity

Data were analyzed using Codebook Thematic Analysis (CTA), as defined by Braun and Clarke [26]: a family of methods that encompasses different approaches for identifying patterns (i.e., themes) within qualitative datasets. Codebook TA was selected because it employs a structured coding method while still allowing researcher reflexivity and flexibility, and because it facilitates a more efficient analytic process and provides greater structure for researchers with limited prior experience in qualitative research [27].

Data analysis began during the transcription of the interview recordings. This approach, combined with repeated review and active reading of the transcripts, enabled the first author to become thoroughly familiar with the data and participants’ narratives. The coding process was conducted using a hybrid approach, as proposed in CTA. Initially, two interviews were coded using a bottom-up (inductive) approach, generating codes without a predefined framework. The initial codes were then reviewed with the second author, who recommended extending the process to a third interview to refine the codes and support the development of a preliminary codebook. Subsequently, all codes were reviewed, refined, and organized into potential themes and subthemes based on similarities, interconnections, and distinctions. The first author developed the thematic maps, which were reviewed and discussed with the second author. This process helped clarify and define themes and subthemes and revealed potential connections among them. The remaining five interviews were then coded using a top-down (deductive) approach, with new codes generated when content was deemed relevant to the study’s objectives and had not been identified in previous interviews. Lastly, themes and subthemes were further refined, resulting in two final themes, which are presented in the Results section.

Finally, to enhance methodological integrity [28] and promote reflexivity, the research team’s positionality and lived experiences that might influence data analysis were considered. The first author is a recent graduate with a master’s degree in psychology who identifies as a cisgender feminist woman and has completed an internship at an organization dedicated to supporting victims of domestic violence. These experiences have informed her analytical and reflexive processes, notwithstanding her limited direct professional experience with domestic violence. Furthermore, she has neither provided nor observed support for trans* individuals. The study was conducted under the supervision of the second author, who has extensive research expertise in LGBTQIA+ issues and identifies as a nonbinary LGBTQIA+ individual, thereby contributing to critical insights and reflexive dialogue that supported a more nuanced and comprehensive analysis. Also, both authors share a research orientation rooted in commitment to social justice and feminist values.

## 3. Results

**Figure 1:**
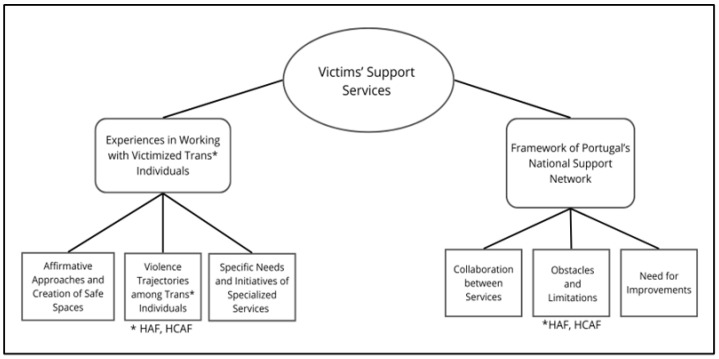
Thematic map depicting participants’ experiences and perceptions.

Data were organized into two themes that reflect participants’ experiences of service provision and perspectives on the work carried out within the National Support Network in cases involving trans* individuals (see Figure 1).

The first theme, (1) Experiences in Working with Victimized Trans* Individuals, encompasses participants’ experiences of providing services to this population, including the Affirmative Approaches and Creation of Safe Spaces (subtheme 1), Violence Trajectories among Trans* Individuals (subtheme 2), and Specific Needs and Initiatives of Specialized Services (subtheme 3). The second theme, (2) Framework of Portugal’s National Support Network, addresses participants’ perceptions of the work within RNAVVD with the trans* population, including the Collaboration between Services (subtheme 1), Obstacles and Limitations (subtheme 2), and Need for Improvements (subtheme 3). Issues related to healthcare and housing were discussed in both themes to highlight how participants approached these areas in an integrated manner, addressing aspects connected to both the experiences of violence faced by trans* individuals and the challenges and limitations of the National Support Network. This emphasizes the needs of this population in these domains. To clarify this, two codes were introduced: Housing-Related Aggravation Factors (HAF) and Healthcare-Related Aggravation Factors (HCAF).

### 3.1. Experiences in Working with Victimized Trans* Individuals

As previously noted, the first theme captures participants’ experiences supporting victimized trans* individuals. When asked how often they had contact with this population, participants from specialized services reported that throughout their professional trajectories, they had supported numerous trans* individuals, particularly young trans* people. Among participants from generalist services, two reported supporting one or more trans* individuals, while one had never professionally worked with trans* individuals. Throughout the interviews, participants discussed the main aspects of their service experiences, which were organized into three subthemes: (i) affirmative approaches and creation of safe spaces; (ii) violence trajectories among trans* individuals; and (iii) specific needs and initiatives of specialized services.

The first subtheme (Affirmative Approaches and Creation of Safe Spaces) synthesizes participants’ perceptions of best practices and key considerations for staff to promote the well-being and protection of trans* individuals. It also addresses the need for professionals in generalist services to assess when referrals to specialized services are necessary and the strategies used in such cases. The second subtheme (Violence Trajectories among Trans* Individuals) concerns the contexts and types of violence against trans* individuals identified as the most prevalent. Issues related to family and intimate partner violence were discussed, as were discrimination and violence within essential services (e.g., healthcare, education, and law enforcement agencies). Finally, the third subtheme (Specific Needs and Initiatives of Specialized Services) concerns participants’ perceptions of the primary needs of victimized trans* people, as shaped by their experiences of violence and structural inequalities. It also addresses initiatives promoted by specialized services to address these needs.

#### 3.1.1. Affirmative Approaches and Creation of Safe Spaces

Throughout the interviews, participants highlighted key attitudes and practices professionals should adopt when assisting trans* individuals, such as adapting language, asking about chosen pronouns and names, raising awareness, referring to specialized services, involving the person, and adapting spaces. The most emphasized need was for professionals to adjust their language practices, to specifically ask pronouns and names, and to respect self-determination in order to build trust. A participant from a specialized service stressed the importance of neutral language, noting that “sometimes it’s not so much about changing an ‘A’ [feminine] or an ‘O’ [masculine] to an ‘E,’ [neutral], but rather saying ‘volunteer person,’ for example” (D.). This participant also highlighted that professionals should acknowledge mistakes, noting that “it’s not difficult to communicate, but our language is extremely gendered, so even when I’m monitoring myself, sometimes things slip through.”

In cases involving trans* minors, professionals from specialized services highlighted several issues that require particular attention. First, one participant warned about the need for caution when sharing information about cases, as doing so may compromise not only privacy but also the safety and well-being of children and adolescents:

One of the things we always have to warn about is that they cannot and should not communicate to legal guardians or caregivers that the person is X or Y without their consent. It’s a direct violation of privacy, and more than that, they risk putting the person—in this case, a child or young person—at risk or even in danger, because the family may react badly (E., specialized service).

The same participant emphasized that even when the young person consents to sharing this information: “There can be problems, and serious ones.” Thus, although involving parents or guardians can, in successful cases, serve as “another protective factor and something that brings resilience to all the processes happening in other contexts”, each case must be carefully assessed to ensure the young person is not placed at greater risk. Other participants emphasized the importance of clarifying doubts and demystifying issues with parents and guardians to help them understand the process the young person is experiencing: “Sometimes they are not supportive or respectful … It’s not because they don’t want to help their children […] it’s because they genuinely don’t know. And sometimes, deconstructing this with parents, especially with trans* kids, is very important” (I., specialized service).

Participants also noted that awareness-raising may be conducted with peers and in schools, provided that such actions are appropriate within the intervention and that the request originates from the person receiving support. As one participant explained, this process “has to do with them, right? And there may be situations in which we are creating contexts that are not desirable for what the person wants for themselves” (E., specialized service). Therefore, this work essentially involves assessing the context in which the person is embedded, identifying intervention needs, and respecting their wishes and desires: “it’s very much about reading the context, understanding whether awareness-raising has already occurred, and not leaving any part of the system the person is part of unattended” (E., specialized service).

Another aspect raised by participants from generalist services concerns the provision of services to trans* individuals in these settings. Participants emphasized that professional teams must assess their capacity and available resources to provide appropriate support to this population. If they determine that they cannot offer the most suitable services, they should refer the person to specialized structures. As one participant noted, “it’s that awareness, as professionals, that we must have, to understand whether we have the competence to move forward with this support” (L., generalist service). It was also emphasized that this decision should be discussed openly and transparently with the person receiving services, allowing them to express their wishes and to make an informed decision about their support process: “always thinking about what’s best for the person, and always giving them the freedom to choose […] the person is an active part of their process of transformation and recovery” (G., generalist service).

One participant from these services reported that, due to frequent collaboration with a specialized structure, their approach in such cases is to contact a professional from the other service and ask them to conduct the first appointment on-site: “so that it doesn’t feel like the person is being pushed around […] They’re seen in our office, and then the next appointments are scheduled at the other service” (C., generalist service). Another strategy mentioned was collaboration between services, in which a professional from the generalist service would be responsible for accompanying the criminal proceedings, while a professional from a specialized service would conduct the therapeutic process. In this way, according to one participant, “we can guarantee that the person will be well supported by another organization, by professionals who may be better equipped to provide the best response for that person” (G., generalist service).

The final aspect addressed the need to adapt communication and physical spaces within services. One participant emphasized the importance of services “knowing how to communicate that they are a safe place, because it’s not enough to be one, you have to be perceived as one” (E., specialized service). Another participant added that services should be “spaces that are very clear and explicit about providing good care to LGBTQIA+ people […] where people can see, ‘okay, I can go there because I know I won’t be discriminated against’” (I., specialized service). Some participants also emphasized that making spaces more LGBTQIA+-friendly goes beyond decorative elements (e.g., rainbow decorations) and is more closely related to “How the team welcomes people” (F., specialized service). They also mentioned the adaptation of institutional spaces: “We try as much as possible to make our spaces and our communication inclusive. We have gender-neutral restrooms, and all the doors indicate that they are restrooms for everyone” (D., specialized service).

#### 3.1.2. Violence Trajectories Among Trans* Individuals

When discussing their professional experiences within victim support services, most participants reported that: “there’s no doubt that trans* people experience greater situations of vulnerability” (B., specialized service), which they attribute to both an increased risk of victimization and barriers to accessing support networks. Participants from specialized services noted that most cases involve multiple forms of violence, primarily perpetrated by family members, though such violence may also occur in other contexts. As one participant explained, this includes “some situations involving schools, and then we also have the occasional case of hate crimes or workplace discrimination” (E., specialized service).

Regarding violence in intimate relationships, professionals noted that although it is less frequent than family violence, they are also experienced by some trans* individuals: “We mostly see family violence, but people also seek us out due to dating violence or even marital violence” (B., specialized service). One participant further warned that dating violence is “a phenomenon that is quite hidden and little talked about” (E., specialized service).

In relation to family violence, participants noted that many of the cases supported by the services involve young trans* individuals who: “are thrown out of home at a very young age. We’re talking about starting at 16, 17, 18 years old” (F., specialized service), which further intensifies housing vulnerability within this population (HAF). Threats of, or actually being, thrown out of home typically occur after individuals disclose their gender identity and/or sexuality to family members, as illustrated in the following example:

I now recall, for example, a specific case we are currently supporting, in which the person is a trans woman who came out to her mother, with whom she lived. The mother said, “Just wait until you turn 18, because for now I can’t kick you out since you’re a minor and that would be a crime, but once you turn 18, you won’t set foot in this house again.” (B., specialized service).

The same participant also noted that many cases involve young trans* individuals whose parents oppose their right to self-determination by prohibiting them from expressing their identity: “Much of this violence occurs through the disrespect of chosen names, the disrespect of pronouns, and even the invalidation of gender identity. That is, not even allowing the person to have a gender expression that aligns with their identity” (B., specialized service). As a result, many young people are forced to mask their gender expression from their parents: “young people who really have to hide, and, for example, trans girls who carry clothes in their backpacks so they can change at school. But then teachers alert the family, and things end up not going well” (B., specialized service).

Another issue raised by participants concerns sexual violence against trans* individuals: “We have more trans* people experiencing sexual violence, whether within intimate relationships, within the family, or outside of it—especially in intimate and family contexts—than cis people” (F., specialized service). The same participant noted that this violence often involves what the literature describes as corrective rape, intended to “change someone’s sexual orientation or gender identity,” and added that “unfortunately, these situations reach us with some frequency.”

This participant also outlined strategies used by perpetrators (both within families and within intimate relationships) to manipulate trans* individuals, such as threatening to ‘out’ them: “If you don’t do X, I’ll tell everyone at your workplace that you’re trans*.” The participant discussed the concept of the “double closet,” where LGBTQIA+ individuals face an additional obstacle in reporting violence due to the fear of having their gender identity or sexual orientation disclosed without their consent: “So there are a number of issues that become compounded, making people often not reporting or not seeking help, either formally or informally, out of fear of being forced out of this double closet.”

Beyond the experiences of victimization within family and intimate relationships, participants also drew attention to episodes of discrimination faced by trans* individuals in essential services: “For example, in interactions with healthcare, education, criminal police bodies, and the justice system” (F., specialized service). Regarding interactions with law enforcement, one participant reported that when accompanying trans* individuals to file complaints, they had encountered situations marked by: “A lot of difficulty in humane treatment, a lot of disrespect” (B., specialized service). They also noted that part of this stems from a lack of understanding among police officers of violence perpetrated against trans* people:

There’s often this idea of, “well, maybe your mother is like this because she doesn’t understand it very well. Maybe you’re not making it easier either. Now you’re talking about pronouns, but this isn’t really a form of violence” […] which are clearly transphobic (B., specialized service).

Participants also mentioned healthcare as one of the contexts in which trans* individuals most frequently experience discrimination and poor professional practices, which increases barriers to access in this area (HCAF). One participant reported that “professionals sometimes seem to lack patience to answer certain questions” (I., specialized service) and that many trans* individuals express fears about “how they will be accepted in these services, or whether there is space for them to explain their gender identity, or whether everything has to be rushed.” Another participant described a case of transphobia in which a doctor, when a trans woman was referred to a hospital, told her: “I’m gay and I also went through a lot to get here. If you’re not capable of going through this, then don’t dress that way, because you’re putting yourself in harm’s way” (D., specialized service).

Finally, professionals also reported cases of discrimination in schools, noting that “most situations are no longer so much between peers, although that still happens. They come from the adult community, school leadership, training staff, teachers, operational assistants… people who hold positions of authority” (E., specialized service). One participant added that “schools are not always prepared to respect chosen pronouns and names […] we have to go to schools to speak with administrators and conduct this type of awareness-raising” (B., specialized service).

#### 3.1.3. Specific Needs and Initiatives of Specialized Services

Participants identified housing, employment, and healthcare as the primary needs of the trans* population, with housing being the most frequently mentioned: “We have many people seeking housing support because, unfortunately, there’s a great deal of discrimination and family violence related to the person’s belonging to the community” (B., specialized service).

Although this is not a problem that affects only trans* people, participants pointed to factors that make it more acute for this population, namely, the additional difficulties in accessing the labor market: “We have several trans* individuals who are experiencing homelessness and engaging in sex work, not by choice but because they are unable to find opportunities for professional integration within the formal labor market” (F., specialized service). One participant discussed these compounded barriers to both housing and employment, stating that: “A trans* person is a victim when looking for housing and perhaps presents as Raquel, while their ID card says Francisco. They are a victim of discrimination in access to employment; the same thing happens with documents” (D., specialized service).

Considering these two interrelated needs, participants from specialized services described initiatives to mitigate some of the difficulties faced by trans* people, including employability networks and transitional housing. The latter refers to independent living apartments primarily intended for young LGBTQIA+ individuals who require support in achieving autonomy, as mentioned by one participant: “What we aim to do is support individuals in leaving situations of violence or, if they have already been removed from that context, to ensure they have the necessary resources” (F., specialized service). The same participant further explained that in the case of young trans* individuals:

We are referring to a particular age group who are legally considered adults but, in reality, are still young people who do not yet know how or have the experience to manage their own lives and care for themselves; therefore, they remain dependent. Our support initially focuses very much on emotional restructuring, because these moments of violence or crisis are often destabilizing […] After that, what we seek to do is design a life project, which may involve finding a job, then finding a room or a home to live in or continuing their education. (E., specialized service).

Similarly, participants mentioned institutional resources promoted by specialized services that aim to assist trans* people in accessing healthcare, including collaborations with specialized healthcare providers and financial support for medical appointments. One participant reported that their institution offers, in addition to the typical services promoted by victim support services (e.g., social, psychological, and legal support): “Endocrinology consultations for trans* or non-binary people who seek these services, to provide an alternative response to the gaps that exist in the National Health Service” (F., specialized service). Another participant mentioned the creation of: “A fund that is used to pay for consultations, mainly in psychiatry and child and adolescent psychiatry, but not only. If there are funds available, we also cover psychology consultations” (E., specialized service). This fund is financed through paid consultations conducted at the institution’s social clinic and supports collaboration with an external network of professionals. It thus serves “as another way of bringing the population closer to professionals who have training and sensitivity in this area, while also mitigating the occasional inability to access mental healthcare due to a lack of financial resources.”

### 3.2. Framework of Portugal’s National Support Network

The second theme explored participants’ perceptions and experiences of working in the National Support Network, particularly regarding issues affecting trans* individuals. This theme is divided into three subthemes: (i) collaboration between services, (ii) obstacles and limitations, and (iii) need for improvements. Notably, all participants were affiliated with services located in large urban centers, such as Porto and Lisbon.

The first subtheme (Collaboration between Services) summarizes participants’ experiences working with other institutions within the National Support Network. It emphasizes factors that promote collaboration (e.g., geographic proximity and prior coordination experience) and barriers (e.g., professionals’ lack of understanding of trans*-related topics and discriminatory attitudes). The second subtheme (Obstacles and Limitations) addresses aspects perceived by participants as hindering trans* people’s access to services, including impacts of broader social problems (e.g., housing crisis) and internal issues within the National Support Network. Finally, the third subtheme (Need for improvements) explores the need for change identified by participants in response to the obstacles described in the previous subtheme. The issues discussed in this subtheme are perceived as crucial for ensuring that services are better equipped to address the needs of the trans* population, including updating mandatory training programs, creating new specialized services, and adopting a more intersectional approach.

#### 3.2.1. Collaboration Between Services

All participants shared their experiences collaborating with other victim support services in the National Support Network. They noted that their professional journey included both successful collaborations and challenges, particularly in securing appropriate responses to cases involving trans* individuals. Overall, participants stated: “When we need to coordinate with other entities, whether LGBTQIA* or not, coordination is usually quite smooth” (F., specialized service). Some participants highlighted that positive coordination experiences often depend on geographic closeness and previous collaboration: “With some entities with whom we already have an established relationship […] from the years of working together, coordination ends up being swift and quite straightforward” (G., generalist service). Additionally, one participant noted that contacts are frequently made: “Directly with specific professionals, in an attempt to speed up communication” (F., specialized service).

Despite that, some participants acknowledged that: “Apart from those entities [LGBTQIA+ services] and perhaps a few others we collaborate with more frequently, there are entities that I know I wouldn’t refer people to” (G., generalist service). One participant noted that the staff knows: “which entities don’t accept [referring to placing trans* people into shelters], and we know which ones do accept, but no one speaks openly about it”, adding that: “There is no open conflict, there is no danger. Institutions and people don’t turn their backs on us, but we know there are barriers” (E., specialized service).

Several participants, especially those working in LGBTQIA+ services, reported encountering cases that revealed a lack of training and knowledge among professionals regarding the specific needs and life trajectories of trans* people: “We still feel that, outside the services specifically for LGBTQIA+ people, there is still a great deal of misinformation and even some prejudice in certain situations” (F., specialized service). One participant mentioned that, when coordinating with generalist services, professionals often have many questions about how to place individuals appropriately when they need to be integrated into other services: “We explain whether it’s a trans woman or a trans man, so that they feel comfortable being placed with people of the same gender identity”, adding that they: “Sense a lot of verbal hesitation, like ‘okay, how are we going to handle this issue?’” (B., specialized service).

Another participant shared an example illustrating this challenge, recounting that when a different organization approached them to assist a trans* individual, they encountered uncertainty from the other professional, who stated:

“She is a woman, but she’s not a woman yet” and I was like… “Huh?” So there was already a great deal of misunderstanding on the other side […] she said “But she’s a man, she hasn’t changed her name or anything” (G., generalist service).

Participants also mentioned cases in which professionals ask questions of a “Questionable nature”, such as “Questioning whether a particular trans* person has undergone gender-affirming surgery”, emphasizing that such information: “Doesn’t change the person’s gender identity, doesn’t change who they are, and doesn’t change their need as a victim to be integrated, for example, into a shelter” (F., specialized service).

Finally, participants reported the following: “there are institutions that don’t respect certain characteristics of some of our service users, namely, for example, in the case of trans* people, their chosen name and pronouns” (I., specialized service). This same participant described an example of such a situation:

They were using the person’s legal name and addressing them, for example, in the masculine or feminine that weren’t the person’s pronouns. On my side, I was discussing the person using their chosen name and correct pronouns. So it felt a bit like a crazy conversation, right? […] It seemed like we were talking about different people and different things (I., specialized service).

#### 3.2.2. Obstacles and Limitations

When discussing their work experiences in victim support services, participants identified factors that pose obstacles to the provision of assistance to victimized individuals. One point raised by most participants concerns the difficulties many services within the network currently face, which, according to one participant, involve “not so much difficulties in coordination with other professionals or entities, but rather difficulties associated with structural, macro-social issues” (L., generalist service). These difficulties are linked to the high volume of assistance requests and the resulting overload of services, which are handling “many people for whom we are unable to respond, nor are we able to refer them to other institutions, because there is simply no capacity to respond” (B., specialized service).

As previously mentioned, many of the cases involving victimized trans* people require social support in accessing healthcare and housing—areas in which participants report experiencing the greatest difficulty in obtaining effective responses from other services (HAF; HCAF). When discussing attempts to coordinate with healthcare services, for example, one participant described the challenges, particularly in psychiatric care (HCAF): “We can make the referral, but the response takes a very long time, and sometimes the call is not returned, or the email is not answered” (G., generalist service).

Regarding housing support, most participants reported that this was the area in which they encountered the most barriers, due to the housing crisis in Portugal and the high volume of requests for support in this domain (HAF): “regardless of the coordination that can be done, we will hardly be able to overcome this difficulty, because it’s beyond the control of any other entity with which we might collaborate” (L., generalist service). One participant also noted how this crisis has affected the network’s shelter responses: “All this difficulty in housing, affecting everyone in the country, means that there aren’t responses to such great need […] this obviously has repercussions for social housing in particular, and more specifically for shelter structures” (F., specialized service).

Beyond these broad issues, participants also discussed specific barriers for trans* people, which include professionals’ lack of specialized training on LGBTQIA+ issues, the scarcity of LGBTQIA+ services, and the unique challenges faced when trying to accommodate trans* individuals in shelters. Regarding the first issue, participants stated that even when professionals strive to do their best when supporting trans* people, many lack the preparation or sensitivity required to meet their needs, as mentioned by one of the participants.

We receive many people who tell us they have already been to other institutions and feel that, even when there is respect, for example, for their name, there isn’t respect for the real needs that a trans* person has, which are different from those of a cis person (B., specialized service).

The same participant also stated that: “On one hand, the lack of knowledge among professionals about gender identity and these issues is understandable” given that they did not receive appropriate training, and as noted by another participant: “They cannot know what they don’t know, what they never had the opportunity to learn, what they didn’t receive training for” (E., specialized service). Participants also mentioned that when referrals to other services are necessary, they often feel as if relying on “luck” to find professionals with adequate training.

In the sense of whether they have training and information about these issues, whether they can monitor and challenge their own prejudices and stereotypes, to adapt inclusive language, use correct pronouns, use their chosen name or the person’s name rather than their deadname. Among many other things” (F., specialized service).

One participant also noted that, despite many situations stemming from a lack of training and familiarity with these topics, there are cases in which professionals repeatedly disregard individuals’ self-determination, even when efforts are made to raise awareness of these issues: “When we refer to the person, we use their chosen name and the pronouns they want. On the other side, they [other professionals] keep calling them by their legal name, using incorrect pronouns. Even in emails and so on” (I., specialized service).

Most participants reported that widespread misinformation about interventions with trans* people persists across services, along with cases of discrimination that lead to their revictimization. This affects the help-seeking behavior of this population, as one participant noted: “People are afraid to seek help from generalist services”, further explaining that for many trans* individuals, seeking formal support: “Is not something trivial like ‘I’ll just go to an institution’. No, it’s “I’ll go to an institution and risk being mistreated, risk not being helped, and even risk that things worsen for me. That’s what many people think” (E., specialized service). These situations cause many LGBTQIA+ people: “Having to seek specific institutions, and those specific institutions are unable to support so many people” (B., specialized service).

On that regard, the second most frequently mentioned obstacle concerns the limited number of LGBTQIA+ services in Portugal and their concentration in major urban centers, as noted by one participant: “Often specialized service are centralized in large cities, and therefore people from other parts of the country are unable to access these specialized services, at least in a face-to-face format” (G., generalist service). This especially impacts trans* individuals living in rural areas: “Because there is a great deal of stigma, many people also have greater difficulty seeking help, because they live in a small community where everyone might find out” (E., specialized service).

Finally, the third obstacle concerns the difficulty in placing trans* people in shelters due to the refusal of many services to accommodate them, as stated by the following participant: “[if a professional] decides to refer a trans* victim to a shelter, for example, any shelter at all, they will face difficulties. Even today, they will. No one will accept them” (C., generalist service). This represents a significant barrier, considering the number of trans* individuals who require this type of support and the limited number of available spaces in the only existing specialized shelter for LGBTQIA+ people in Portugal. Some participants mentioned cases where trans* individuals refused to contact the National Social Emergency Line (responsible for supporting placements), justifying this decision: “‘Because they will put me in a shelter for men, and I don’t want to go’ or ‘that already happened, and I won’t call again because I don’t want that to happen again.’” (I., specialized service). One participant also mentioned cases involving the placement of young trans* individuals, which demonstrate efforts by professionals to meet the needs of this population, while also revealing ongoing difficulties:

There was a sensitivity like, okay, you are a trans* person, we won’t place you with either male or female population. We will place you […] as a trans woman, in the male wing, but in an individual room. […] after that, the shared spaces still have to be with the male population, even though she is a trans woman. (B., specialized service).

Participants also highlighted concerns in what is described as: “The greatest difficulty for me, and it happens somewhat more significantly with the trans* population, particularly women, is issues of severe mental health. Because these people are unable to remain in any type of service” (E., specialized service). Addressing this issue, one participant described an example that illustrates the challenges faced when attempting to place trans* individuals with this specific intersection of vulnerabilities: “This person exhausted all available options. They were referred to the only specialized LGBTQIA+ response, it didn’t work out. They were referred to another shelter solution, it didn’t work out […] institutions are not prepared for these people” (D., specialized service).

#### 3.2.3. Need for Improvements

When discussing the need for changes and improvements within the National Support Network, most participants emphasized that mandatory training must include specific guidance on interventions with the LGBTQIA+ community for all personnel working in the field. One participant also noted that professionals currently engaged in the services should have their training updated: “You take the course when you want to join the services, but things change, laws change, the world changes. It would be good if at least the network could schedule moments of specific training for these updates” (D., specialized service).

Regarding the number of specialized LGBTQIA+ services, some participants stated that new structures must be created to address the needs of this population effectively: “I think it’s about creating more responses, for example, more emergency shelters for the LGBTQIA+ population and also decentralizing the services” (I., specialized service). Other participants suggested that the focus should be on equipping generalist services to work with this population: “I believe there shouldn’t be specialized structures; rather, each service should have one or two trained professionals, or perhaps everyone […] to ensure wider national coverage” (C.; generalist service). One participant noted that specialized services should act as support for LGBTQIA+ needs until the network can fully address them:

We function only as a crutch, a way to fill a gap that isn’t yet ideal: in the long term, anyone who seeks help anywhere will, at minimum, receive proper treatment, have access to the resources they need, and be protected and safeguarded (E.; specialized service).

Participants also emphasized the need to develop shelter solutions to accommodate trans* individuals in need of placement, whether by creating new specialized structures or adapting existing facilities: “It’s the network’s responsibility to ensure that either there are more specialized shelters for LGBTQIA+ people—currently, we have only one—or that there are trained teams in other shelters” (D., specialized service). One participant emphasized that shelters should be inclusive for anyone needing placement, rather than categorizing people into separate groups: “Shelters should be transversal for anyone who needs to be placed there. The truth is this is not the case. We still put people into little boxes: these go here, those go there” (C.; generalist service). Another participant stressed that the key step is to discuss these collectively within organizations to develop effective, implementable solutions:

There are important underlying aspects to discuss, and I am not an advocate of top-down measures without explaining why or without giving people the opportunity to be heard […] I’m not saying it will solve everything, but I think if we can discuss the problems and difficulties together, and then in a sense as “So how are we going to solve this?”, “What can we do?” It’s much more productive (E., specialized service).

Lastly, several participants highlighted the importance of services considering the intersectionality of victimized individuals, with one participant stating that: “it’s about understanding that a victim is not just a standard profile, not just a white cis woman. We also feel a lot of devaluation of people, for example, black women. Black women are not as heard” (B., specialized service).

## 4. Discussion

This study aimed to explore the experiences of professionals working with victimized trans* individuals, as well as their perspectives on service provision for this population within Portugal’s National Support Network for Domestic Violence Victims. Importantly, the findings reflect professionals’ interpretations of trans* individuals’ experiences rather than direct accounts from trans* people. As such, they provide insight into how institutional responses are perceived and enacted, rather than how they are experienced.

Professionals described multiple factors that, from their perspectives, exacerbate the psychosocial vulnerabilities of trans* people—including violence within families and within intimate relationships, systemic discrimination, and inequalities in access to essential services. Their accounts suggest that these vulnerabilities are not produced solely through interpersonal violence but also mediated through institutional practices. For example, reports of the minimization of identity-based violence in interaction with the police forces, barriers to gender-affirming care in healthcare settings, and difficulties in shelter placement illustrate how services are often organized around implicit cisnormative assumptions. In this sense, structural cisnormativity operates not only as a broader social condition but as a set of everyday institutional practices that shape recognition, access to resources, and protection for trans* individuals.

When describing their experiences working in victim support services, participants reported encountering a high prevalence of trans* people experiencing family violence (e.g., being thrown out of home, disrespect of pronouns, prohibiting gender expression, etc.), particularly among young trans* individuals. As Ramalho et al. [29] note, when families perceive the gender expression of trans* people as socially inappropriate, they often impose restrictions and punish behaviors that don’t conform to gender norms and stereotypes. Given the central role of families in the biopsychosocial well-being of young trans* people and the association of their absence with clinically significant distress [18,30], initiatives mentioned by participants, such as providing parents clarification and demystifying trans* affirmation processes, are essential to safeguarding this population’s well-being.

Regarding intimate partner violence, professionals reported encountering fewer cases involving trans* people compared to cases of family violence. Nevertheless, recent international research [15,16,17] indicates that trans* individuals are at equal or higher risk of victimization in these contexts than cisgender people. Building upon the Gender Minority Stress Model [30], participants’ accounts may reflect how structural and interpersonal stressors—such as discrimination, anticipated rejection and the need to manage identity disclosure—can shape help-seeking behaviors and reduce the likelihood of reporting intimate partner violence; especially if the person fears being ‘outed’ and having their gender identity or sexual orientation disclosed without their consent [7].

Recent mixed-methods Portuguese studies on violence and discrimination against LGBTQIA+ people [6,7] corroborate the main findings of the present study: domestic violence against this population often begins early on, even in childhood, and persists throughout their life course [7]. Saleiro et al. [6] also suggest that violence against LGBTQIA+ people is mostly perpetrated within families, primarily by parents, and that gender-identity-related issues may prompt earlier requests for support from specialized services.

By adopting a qualitative methodology and focusing on the experiences of professionals who support victimized trans* people, this study contributes to a more detailed understanding of how professionals perceive the impacts of domestic violence for this population. For instance, participants’ accounts demonstrate how structural cisnormativity across different sectors, such as housing, employment, healthcare and victim support services, acts as additional barriers that often compromise this population’s autonomy processes, especially that of trans* youth. These findings suggest that violence cannot be understood solely through gender identity, but through the interaction between gender marginalization, age-related dependency, housing precarity and labor exclusion.

In Portugal, family often serves as the primary support system for young people, given the country’s strong family-oriented values, high youth unemployment rates, and limited government assistance for youth and young adults [31]. Consequently, the absence of family support, combined with rising housing costs, often hinders access to appropriate housing and to independence.

As most participants noted, these issues are compounded for trans* people by additional barriers to entering the labor market: Baptista’s [32] study shows that trans* people in Portugal experience higher unemployment rates, job insecurity, and workplace discrimination, such as refusal to use their social name, denial of access to changing rooms, and role reassignments to reduce client exposure. These accounts illustrate how gender identity often intersects with class and economic vulnerabilities, which further constrain access to stable employment and housing, thereby reinforcing cycles of social and economic marginalization among trans* people. Therefore, initiatives such as those mentioned by participants (e.g., employability networks and autonomy apartments for young LGBTQIA+ people) are essential resources that support trans* people on their paths toward independence.

As previously mentioned, another context that often represents this systemic neglect against trans* people is healthcare service. In the present study, participants mentioned numerous instances where trans* people reported being discriminated against by healthcare providers, and that they perceived a marked lack of sensitivity and professional training. These barriers may demonstrate the effects of cisnormativity within healthcare systems, where protocols, assumptions, and training frameworks are not designed to accommodate gender diversity. This aligns with previous research, which shows that prejudice and lack of specialized training among healthcare providers hinder trans* people’s access to appropriate care [33]. Discriminatory practices include the use of prejudiced language, denial or discouragement of gender identity exploration, refusal of care, interruption of treatment, and ridicule of trans* service users [34].

Participants also highlighted systemic constraints within the National Health Service in Portugal, including referral bottlenecks and service overload. While isolated initiatives were described as positive, participants emphasized that structural limitations, particularly human and financial resource shortages, constrain their effectiveness. In this sense, access barriers are not only organizational but embedded within broader structural conditions that shape service availability and responsiveness. Therefore, public policies must be created and properly implemented to ensure that trans* people can access the healthcare they require [35].

Regarding access to victim support services, professionals indicate that trans* people face multiple, intersecting barriers that hinder service use. Some of these barriers, such as fear of discrimination and limited availability of specialized, culturally competent services, have been documented in previous research (e.g., [20,22]). Extending this literature, the present study highlights the geographic concentration of specialized services in large urban centers as an additional structural barrier. Since the experiences of trans* people in rural and nonmetropolitan regions of Portugal remain under-researched, future studies are needed to better understand how services provided by the National Support Network outside Porto and Lisbon meet their specific needs and what are the barriers that they face.

Another significant barrier identified in participants’ accounts concerns access to shelter services. As most participants noted, Portugal has a very limited number of specialized responses for the LGBTQIA+ population, including only one specialized emergency shelter, alongside the absence of specialized long-term shelters. While other responses exist, such as autonomy apartments, these serve a different purpose and do not provide the same level of immediate protection.

Participants further highlighted that, beyond the refusal of some generalist services to accommodate trans* individuals, a broader issue lies in the lack of preparedness and competence of many generalist services to adequately respond to this population’s needs. These constraints contribute to the overburdening of the only specialized emergency shelter and limit its ability to accommodate new people in need [24]. These challenges are further intensified when gender identity intersects with other vulnerabilities, such as mental health needs, creating situations in which individuals do not fit existing service models.

Since shelter services are essential for both enhancing the safety of victimized individuals and supporting their autonomy, the absence of accessible responses for trans* individuals further entrenches their social entrapment [36]. In this sense, lack of access to shelter is not merely a service gap but it reflects broader structural limitations in how protection systems are designed and implemented.

Importantly, participants’ accounts revealed a tension, rather than a consensus, between two approaches: the expansion of specialized LGBTQIA+ shelters versus strengthening inclusivity and responsiveness within mainstream generalist shelters. While specialized services were perceived as safer and more culturally competent, participants also questioned whether reliance on them may unintentionally reinforce the exclusion of trans* people from generalist services. Conversely, although generalist services may promote broader accessibility, participants questioned whether they currently possess the institutional capacity and training required to provide affirmative support. Reports of misgendering, inappropriate placement decisions, and uncertainty in handling trans*-specific cases suggest that inclusivity is not yet structurally embedded within these systems, but rather dependent on individual staff awareness and goodwill.

This tension reflects a broader structural dilemma within service provision: whether to prioritize the development of specialized, targeted responses or to transform existing systems to ensure universal inclusivity. Participants’ accounts suggest that these approaches should not be understood as mutually exclusive. Instead, the findings point to the need for a dual strategy that combines the expansion of specialized services, particularly to address urgent safety needs, with systemic efforts to embed gender-affirming practices across generalist services.

These challenges are closely linked to not only gaps in professionals’ training but also to broader institutional failures in integrating gender diversity into domestic violence responses. Participants’ accounts suggest that the absence of systematic training contributes to the reproduction of cisnormativity assumptions and inconsistent practices across services—such as uncertainty regarding appropriate language, inconsistent recognition of gender identity, and difficulties responding to cases involving trans* people within procedures primarily developed for cisgender women. This indicates that institutional responses to domestic violence continue to be shaped by gendered assumptions that position cisgender experiences as normative and structurally marginalize trans* people within support systems.

Previous studies [7,13,25] with a similar profile of participants substantiate these findings: there is a marked lack of specialized training in LGBTQIA+ issues and a sense of unpreparedness to provide support to this population in victim support and Public Administration services. Despite the recent reformulation of the mandatory training program in the area, which included specifics for assisting vulnerable groups (e.g., LGBTQIA+ people, older people, individuals with severe mental health issues), the delivery of these contents appears insufficient. Given that professional competence in working with the trans* population is specific and relies on experiences and training beyond those required for interventions with LGB populations [19], the results indicate a need to address these content areas in greater depth and to reassess mandatory training.

Overall, this study contributes to understanding how professionals interpret and respond to the needs of trans* individuals within the Portuguese support system. Rather than reflecting direct accounts of lived experience, the findings illuminate how such experiences are mediated through institutional practice, shaped by structural cisnormativity and differentiated through intersecting axes of inequality across domestic violence services, housing, employment, and healthcare sectors. By situating these accounts within broader structural frameworks, the study highlights how violence and vulnerability are not only interpersonal but institutionally shaped, revealing the role of service systems in reproducing or mitigating inequality across sectors.

### Limitations and Suggestions for Further Research

This study has limitations that must be acknowledged. First, although the term trans* has been used as an umbrella term, this may not fully align with participants’ perspectives. As a result, the findings may not reflect the experiences of support for individuals whose gender identities extend beyond the binary (e.g., non-binary, genderfluid, and agender individuals). Future research should examine the specifics of working with this population. Furthermore, although intersex individuals were not the primary focus of this study, some participants highlighted them as an overlooked and invisibilized population. Therefore, future studies should explore and recognize their experiences within the Portuguese context.

Second, the use of non-probabilistic sampling, while essential for engaging professionals willing to discuss issues related to supporting trans* individuals, also raises concerns about potential self-selection bias, given that all interviewed participants endorsed trans* rights advocacy. Furthermore, all participants were employed in services located in large urban centers, with only three working in generalist services. This distribution likely shaped the findings towards more informed, trained and affirmative perspectives, while offering a less comprehensive account of the challenges, resistance or lack of capacity that may be more common in generalist services.

Consequently, future research should map the experiences of support provided to trans* individuals across RNAVVD’s services, particularly in rural areas, to attain a more accurate understanding of service delivery nationwide. Similarly, a comprehensive evaluation of mandatory domestic violence training (TAV) is needed to assess the implementation of content related to interventions with the trans* population.

## 5. Conclusions

Despite advances in LGBTQIA+ rights in Portugal and growing public and academic interest in gender diversity, the Portuguese literature on the lived experiences of trans* people remains limited. Therefore, the findings from this study provide important insights that can inform practices within victim support services and Public Administration, addressing the violence trajectories, specific needs, and barriers to accessing RNAVVD’s services among victimized trans* individuals. Furthermore, the results highlight initiatives undertaken by specialized services to meet their psychosocial needs.

Findings highlight how structural cisnormativity operates across housing access, employment, healthcare and victim support services, shaping unequal access to protection and support. Participants consistently described institutional practices organized around cisnormative assumptions, which collectively constrain service accessibility for trans* individuals. In terms of policy and practice implications, the results suggest three interconnected areas for improvement.

First, although domestic violence training (TAV) formally includes LGBTQIA+ content, participants’ accounts highlight persistent gaps concerning the intervention with victimized trans* individuals. Therefore, there is a need to reassess mandatory training and promote continuous professional development to ensure that generalist services are equipped to support this population effectively. This may include more practice-oriented and scenario-based training focused on gender-affirming interventions, the careful use of adequate language, and the specific dynamics of domestic violence affecting trans* individuals.

Second, regarding shelter services, the findings point to not only a shortage of LGBTQIA+ specialized shelters but also to inconsistencies in how generalist services manage admission and safety for trans* individuals. This indicates that beyond expanding specialized responses, there is a need for clearer, standardized protocols to guide inclusive practice across shelter systems and reduce discretionary exclusion. There is also a need to adapt shelter spaces to accommodate the specific needs of trans* individuals, including ensuring privacy, safety, and access to gender-affirming healthcare, such as hormonal treatments.

Lastly, public policies must address the broader structural factors that increase trans* people’s vulnerability, moving away from fragmented or sector-specific solutions. A coordinated, cross-sector approach should be adopted, integrating housing, employment, healthcare, and social protection systems. This recognizes that domestic violence and intimate partner violence are deeply connected to structural inequalities. In practice, responses to violence should go beyond immediate protection measures, providing access to stable housing, employment opportunities, timely and competent healthcare, and sufficient income support. These elements are essential for fostering long-term safety and independence.

In sum, the findings from this study suggest that public policies should aim not only to develop more inclusive social responses but also to strengthen mechanisms that guarantee the effective enforcement of trans* people’s rights. Authorities need to consider the unique needs of this group when developing and implementing policies to ensure that essential services can meet their psychosocial needs. Ultimately, the results emphasize the need for a holistic, intersectional approach, not only within victim support services but also across sectors of society.

## Data Availability

The data presented in this study are available on request from the corresponding author due to concerns over protecting participants’ anonymity.

## References

[1] Moleiro C., Alarcão V., Neves L.R. (2023). Mapping transgender studies in Portugal: A systematic search and narrative review. J. Gend. Stud..

[2] Neves S., Borges J., Ferreira M., Correia M., Sousa E., Rocha H., Silva L., Allen P., Vieira C.P. (2023). A literature review on violence and discrimination against trans people in Portugal: Are we still living in a dictatorship?. Sexualities.

[3] Abreu M.S., António R., Moleiro C. (2025). Mind the gap! LGBTQI+ population’s perceptions of discrimination and of legal innovation. Sex. Res. Soc. Policy.

[4] European Union Agency for Fundamental Rights LGBTIQ Equality at a Crossroads: Progress and Challenges: EU LGBTIQ Survey III. https://fra.europa.eu/sites/default/files/fra_uploads/fra-2024-lgbtiq-equality_en.pdf.

[5] ILGA EUROPE Annual Review of the Human Rights Situation of Lesbian, Gay, Bisexual, Trans and Intersex People in Europe and Central Asia 2025. https://www.ilga-europe.org/files/uploads/2025/02/ILGA-Europe-Annual-Review-2025.pdf.

[6] Saleiro S.P., Ramalho N., Menezes M.S., Gato J. (2022). Estudo Nacional Sobre as Necessidades das Pessoas LGBTI e Sobre a Discriminação em Razão da Orientação Sexual, Identidade e Expressão de Género e Características Sexuais.

[7] Neves S., Ferreira M. (2023). Trajetórias de Vida de Pessoas Lésbicas, Gays, Bissexuais, Trans e Intersexo (LGBTI) Vítimas de Violência Doméstica: Principais Resultados.

[8] Manita C., Ribeiro C., Peixoto C. (2009). Violência Doméstica: Compreender Para Intervir, Guia de Boas Práticas Para Profissionais de Saúde.

[9] Moleiro C., Pinto N., Oliveira J.M., Santos M.H. (2016). Violência Doméstica: Práticas No Apoio a Vítimas LGBT: Guia de Boas Práticas Para Profissionais de Estruturas de Apoio a Vítimas.

[10] World Health Organization (2021). Violence Against Women Prevalence Estimates, 2018: Global, Regional and National Prevalence Estimates for Intimate Partner Violence Against Women and Global and Regional Prevalence Estimates for Non-Partner Sexual Violence Against Women. https://www.who.int/publications/i/item/9789240022256.

[11] European Institute for Gender Equality Gender Equality Index 2024: Tackling Violence Against Women, Tackling Gender Inequalities. https://eige.europa.eu/publications-resources/publications/gender-equality-index-2024-tackling-violence-against-women-tackling-gender-inequalities?language_content_entity=en.

[12] Governo da República Portuguesa (2025). Relatório Anual de Segurança Interna (RASI 2024). XXIV Governo Constitucional. https://www.portugal.gov.pt/pt/gc24/comunicacao/documento?i=relatorio-anual-de-seguranca-interna-rasi-2024.

[13] Furman E., Barata P., Wilson C., Fante-Coleman T. (2017). “It’s a gap in awareness”: Exploring service provision for LGBTQ2S survivors of intimate partner violence in Ontario, Canada. J. Gay Lesbian Soc. Serv..

[14] Rogers M. (2019). Challenging cisgenderism through trans people’s narratives of domestic violence and abuse. Sexualities.

[15] Langenderfer-Magruder L., Whitfield D.L., Walls N.E., Kattari S.K., Ramos D. (2016). Experiences of intimate partner violence and subsequent police reporting among lesbian, gay, bisexual, transgender, and queer adults in Colorado: Comparing rates of cisgender and transgender victimization. J. Interpers. Violence.

[16] Peitzmeier S.M., Malik M., Kattari S.K., Marrow E., Stephenson R., Agénor M., Reisner S.L. (2020). Intimate partner violence in transgender populations: Systematic review and meta-analysis of prevalence and correlates. Am. J. Public Health.

[17] Closson K., Boyce S.C., Johns N., Inwards-Breland D.J., Thomas E.E., Raj A. (2024). Physical, sexual, and intimate partner violence among transgender and gender-diverse individuals. JAMA Netw. Open.

[18] Cardona M.J., Pinto N., Moleiro C. (2022). Experiences of families of trans and gender diverse youth in Portugal within an ecological systems framework. Port. J. Soc. Sci..

[19] Moleiro C., Raposo C.S., Moita G., Pereira H., Gato J., Silva M., Neves S. (2017). Guia Orientador da Intervenção Psicológica com Pessoas Lésbicas, Gays, Bissexuais e Trans (LGBT).

[20] Marrow E., Malik M., Pantalone D.W., Peitzmeier S. (2024). Power and control, resistance and survival: A systematic review and meta-synthesis of the qualitative literature on intimate partner violence against transgender individuals. Soc. Sci. Med..

[21] Dufour G.K., Gerhardt E., McArthur J., Ternes M., Shackelford T.K. (2023). Help-seeking behavior and domestic violence. Encyclopedia of Domestic Violence.

[22] Calton J.M., Cattaneo L.B., Gebhard K.T. (2016). Barriers to help seeking for lesbian, gay, bisexual, transgender, and queer survivors of intimate partner violence. Trauma Violence Abus..

[23] Sousa E., Neves S., Ferreira M., Topa J., Vieira C.P., Borges J., Costa R., Lira A. (2023). Domestic Violence against LGBTI People: Perspectives of Portuguese Education Professionals. Int. J. Environ. Res. Public Health.

[24] Allen P., Neves S., Ferreira M. (2022). O papel das estruturas de atendimento, acolhimento e abrigo na intervenção junto de vítimas de violência doméstica LGBTI. Investigação e Prática: Abordagens Interdisciplinares Sobre a Saúde e o Bem-Estar das Pessoas LGBTI+.

[25] Tesch B., Bekerian D.A. (2015). Hidden in the margins: A qualitative examination of what professionals in the domestic violence field know about transgender domestic violence. J. Gay Lesbian Soc. Serv..

[26] Braun V., Clarke V. (2019). Reflecting on reflexive thematic analysis. Qual. Res. Sport Exerc. Health.

[27] Braun V., Clarke V., Hayfield N., Terry G., Liamputtong P. (2019). Thematic analysis. Handbook of Research Methods in Health Social Sciences.

[28] Levitt H., Wertz F., Motulsky S., Morrow S. (2017). Recommendations for designing and reviewing qualitative research in psychology: Promoting methodological integrity. Qual. Psychol..

[29] Ramalho N.A., Silva A.C., Santos B.M. (2015). A intervenção social com populações “desassistidas” em contexto de rua: O caso do projeto ‘Trans-Porta’. Interv. Soc..

[30] Testa R.J., Habarth J., Peta J., Balsam K., Bockting W. (2020). Development of the Gender Minority Stress and Resilience Measure. Psychol. Sex. Orientat. Gend. Divers..

[31] Gato J., Leal D., Seabra D. (2020). Quando a casa não é um porto seguro: Efeitos da pandemia COVID-19 em jovens de minorias sexuais e de género em Portugal. Psicologia.

[32] Baptista J.D. (2024). Integração de Pessoas Trans No Mercado de Trabalho em Portugal: Perspetivas Sociais, Organizacionais e Individuais. Ph.D. Thesis.

[33] Pinto N., Moleiro C. (2012). As experiências de cuidados de saúde de pessoas transexuais em Portugal: Perspetivas de profissionais de saúde e utentes. Psicologia.

[34] Rodrigues L., Soares M., Nogueira C. (2021). Psychomedical Interventions with Transgender People in Portugal and Brazil: A Critical Approach. Int. J. Environ. Res. Public Health.

[35] Parra-Villarroel J., Rodrigues L., Nogueira C. (2023). Acesso a cuidados de saúde para adolescentes transgénero: Situação atual e desafios no Chile e em Portugal. Análise Soc..

[36] Greenberg K. (2012). Still hidden in the closet: Trans women and domestic violence. Berkeley J. Gend. Law Justice.

